# A prospective study of neurofibromatosis type 1 cancer incidence in the UK

**DOI:** 10.1038/sj.bjc.6603227

**Published:** 2006-06-20

**Authors:** L Walker, D Thompson, D Easton, B Ponder, M Ponder, I Frayling, D Baralle

**Affiliations:** 1 Department of Medical Genetics, Addenbrookes Hospital, Hills Road, Cambridge CB2 2QQ, UK; 2 Cancer Research UK Genetic Epidemiology Unit, Strangeways Research Laboratory, Cambridge CB1 8RN, UK; 3 Cancer Research UK Department of Oncology, Hutchison/MRC Research Centre, Cambridge CB2 2XZ, UK; 4 Faculty of Social and Political Sciences, Centre of Family Research, University of Cambridge, Free School Lane, Cambridge CB2 3RF, UK; 5 Medical Genetics Service for Wales, Institute of Medical Genetics, University Hospital of Wales, Cardiff CF14 4XN, UK

**Keywords:** NF1, cancer incidence, prospective

## Abstract

Neurofibromatosis type 1 (NF1) is an autosomal dominant condition affecting around one in 3000 live births. The manifestations of this condition are extremely variable, even within families, and genetic counselling is consequently difficult with regard to prognosis. Individuals with NF1 are acknowledged to be at increased risk of malignancy. Several studies have previously attempted to quantify this risk, but have involved relatively small study populations. We present prospective data from 448 individuals with NF1 with a total of 5705 years of patient follow-up. These data have been collected via the UK NF1 association for patients. Demographic information on the affected individuals was cross-referenced with UK cancer registry data by the UK Office of National Statistics. The overall risk of cancer was 2.7 times higher in this cohort of NF1 patients than in the general population (95% confidence interval (CI) 1.9–3.7). The cumulative risk of a malignancy by age 50 years was 20% (95% CI 14–29%); beyond this age, the risk of cancer was not significantly elevated (*P*=0.27). The most frequent types of cancer were connective tissue (14% risk by age 70, 95% CI 7.8–24%) and brain tumours (7.9, 95% CI 3.9–16%). There was no statistically significant excess of cancers at other sites (*P*=0.22).

Neurofibromatosis type 1 (NF1) (OMIM 162200), formerly called von Recklinghausen disease, is an autosomal dominant condition affecting one in 3000 live births (Huson *et al*, 1989). Until recently, diagnosis of this condition has only been made according to well-established clinical diagnostic criteria (Gutmann *et al*, 1997). Following the isolation of the gene responsible in 1990 (Viskochil *et al*, 1990; Wallace *et al*, 1990), molecular diagnosis can now be made. From the diagnostic criteria, it is clear that NF1 may involve almost every organ system in the body, with considerable inter-familial and intra-familial variation in manifestations of the condition (Huson SM). Neurofibromatosis type 1 can remain static or be rapidly progressive, but it is not currently possible to predict outcome for individuals. For these reasons, genetic counselling in NF1, with regard to its non-malignant manifestations, is difficult. In addition, the spectre of an increased risk of malignancy in these individuals causes great concern for families.

This increased cancer risk has been variously estimated by several groups around the world, but has not been clearly delineated. Previous data have indicated overall additional malignancy risks in NF1 of between 5 and 15% (Sorensen *et al*, 1986; Huson *et al*, 1988; Baptiste *et al*, 1989; Matsui *et al*, 1993; Stiller *et al*, 1994; Friedman and Birch, 1997; Zoller *et al*, 1997; Evans *et al*, 2002), and these figures are commonly used when counselling patients; therefore, a more specific risk based on a larger study population would be useful. In addition, the advent of surveillance techniques for early detection and subsequent treatment of malignancy suggested that a more detailed analysis of the question of malignancy risk in NF1 was needed.

To address this question, we now present prospective data concerning the incidence and type of malignant tumours in a cohort of 448 individuals with NF1.

## MATERIALS AND METHODS

Families were recruited where at least one member was affected by NF1 via the UK patient support group for this condition (previously known as LINK, now called the Neurofibromatosis Association UK). These families completed a demographic data collection form detailing which family members were affected by NF1. All the patients recruited for the study had been diagnosed with NF1 by either a hospital doctor or a general practitioner, and the forms submitted by the families were reviewed by the authors to ensure that each patient had a high likelihood of a diagnosis of NF1.

Data on these individuals were submitted to the UK Office of National Statistics, where cross-referencing with UK cancer registries occurred. We could thus obtain information about these individuals with regard to mortality and cancer incidence.

Ethical approval was obtained at the beginning of the project Protocol No: 511, Royal Marsden Hospital.

### Description of the cohort

A total of 464 individuals with a clinical diagnosis of NF1 were initially included in the study cohort. Individuals came from 299 different families, with a median number of individuals per family of one (189 families with one member, 68 with two, 31 with three, nine with four and two families with five members). The cohort contained 231 male (49.8%) and 233 female (50.2%), with dates of birth between 1918 and 1990 (median birth year=1965, inter-quartile range (IQR)=1949–1978).

Participants were followed up prospectively, starting at the date that the patient's ‘form’ was returned to the study coordinator, which was between 1st July 1990 and 3rd July 1991. Only the first malignancy in each patient was considered, excluding non-melanoma skin cancers and benign tumours, and follow-up continued until the earliest first malignant cancer, death, eightieth birthday or 1st April 2004, as applicable. Thirteen patients were excluded from the cohort because their first cancer diagnosis was before their form being returned (cases diagnosed between 1976 and 1989), and three others were excluded because they had died before their form was returned by their next of kin.

With these exclusions, the cohort used in the main analysis contained 448 NF1 patients (221 male and 227 female), followed for a median of 13.6 years (range=2.4 months to 13.8 years; 87% of subjects accrued at least 13 years of follow-up), with a total of 5705 person-years of follow-up. This was a predominantly young cohort of NF1 patients, with a median age at entry of 26 years (range=7 months to 72 years). Forty four per cent of the cohort was younger than 20 years of age at entry.

Of the 413 NF1 patients who did not develop a malignancy during the follow-up period, 20 were censored at their date of death (median age at death=45.9 years, IQR=35.8–62.1 years). Three others reached their eightieth birthday before the end of the period, and the remaining 389 patients were censored at 1st April 2004 (median age at censoring=35.9 years, IQR=24.5–52.9).

### Statistical methods

Standardised incidence ratios (SIR) were used to compare the cancer incidence in NF1 patients with that expected in the general population. (Note that all SIRs presented here are ratios of observed to expected numbers of cases, not multiplied by 100.) Expected numbers of cancers in each individual were based on the age, sex and calendar-period specific incidence rates given for England and Wales in Cancer in Five Continents Volumes VII and VIII (Parkin *et al*, 1997, 2002) using the PYEARS program (Coleman *et al*, 1989). The rates published in Volume VIII (7) are the most recent available and cover the period 1988–1992; these were assumed to apply up until the end of the period of follow-up. The 95% confidence intervals (CIs) were derived as exact confidence limits for a Poisson mean (Breslow and Day, 1997), and two-sided *P*-values were also derived from the Poisson distribution.

The analysis was repeated excluding the five cancer cases that were reported on death certificates, but not by the cancer registry. Standardised incidence ratios were also estimated separately for affected individuals who were below age 50 years and for those aged 50 years or older (the median age at first cancer diagnosis in the cohort was 47 years).

Cumulative risks of cancer in patients were estimated by applying the estimated SIRs for the two age groups to the population rates for England and Wales (1992–1997) (Parkin *et al*, 2002).

The SIRs presented here ignore the non-independence of cancer incidence in relatives from the same family, which would exist if mutations in genes other than NF1 act to modify the cancer risk in patients, or if particular NF1 mutations confer different risks of cancer. In the absence of pedigree information, we were not able to address this issue. Nevertheless, the assumption of independence is unlikely to have introduced an important bias, as the majority of families had only one (63%) or two (24%) members in the final cohort. Two families had more than one patient with a cancer case included in the analysis; both had one member with a brain tumour and one member with a malignant peripheral nerve tumour.

## RESULTS

During the period of follow-up, 36 malignant tumours were observed in the 448 individuals in the cohort (i.e. 8.0% of the cohort had a malignancy during the follow-up period), compared with 13.4 expected; SIR=2.7 (1.9–3.7), *P*<0.0001. These are listed in Table 1 and Appendix A. Given the number of cancers expected, the study had a 90% power to detect an SIR of at least 2.1 at the conventional 5% significance level. Twenty-three of the tumours were in female (SIR=3.07, (1.9–4.6), *P*<0.0001) and 13 were in male (SIR=2.2 (1.2–3.8), *P*=0.015). The difference in the incidence ratios between the sexes was not significant (*P*=0.34).

The most commonly observed types of cancer were connective tissue tumours (11 cases) and brain tumours (seven cases). Together, these comprise half of the tumours reported. These types of tumour are extremely rare in the general population; hence, the ratios of observed/expected tumours in these two categories are highly significant. The excess risk for both types of tumour were evenly distributed between the sexes; there were six connective tissue tumours in male (SIR=128 (46.9–279), *P*<0.0001) and five in female (SIR=116 (37.6–270), *P*<0.0001), *P*=0.88 for the difference between the male and female SIRs. Similarly, there were four brain tumours in male and three in female; SIR=23.8 (6.5–60.9) *P*=0.0001 and SIR=21.2 (4.4–61.8) *P*=0.0009, respectively, *P*=0.88 for the difference. Of the brain/CNS tumours detected by this study, astrocytomas were the most common histological type, with cranial nerve gliomas also well represented.

Excluding connective tissue and brain/CNS tumours, the risk of cancer was not significantly higher than expected in the general population (SIR=1.4 (0.8–2.2) *P*=0.22). There was actually a nonsignificant deficit in males (three cancers, SIR=0.53 (0.1–1.5), *P*=0.37), but some evidence of an excess in females (15 cancers, SIR=2.0 (1.2–3.4) *P*=0.017). The 15 tumours in females comprised two colorectal cancers, four at unknown sites and five breast cancers, along with single case each of lip, ovarian, bladder and parathyroid cancer, none of which represented a statistically significant increase in risk. This sex ratio replicates that of other groups reporting malignancy in NF1 (22), but may be partly explained by the specific types of cancer observed.

The overall cancer risk was significantly increased in patients younger than 50 years of age; SIR=6.5 (4.1–9.8), *P*<0.0001 (Table 2a). The connective tissue tumours and brain/CNS tumours were both significantly more frequent than in the general population (SIR=132 (48.6–289) *P*<0.0001 and SIR=47.8 (19.2–98.4) *P*<0.0001, respectively). The overall cancer risk excluding these two groups of sites was also significantly elevated. The risk of breast cancer was significantly higher in this age group than in the general population; SIR=4.0 (1.1–10.3), *P*=0.037. Of five cases of breast cancer diagnosed in the cohort, four were before age 50 years (ages 39, 43, 46 and 48 years), and the fifth case was diagnosed at age 56 years. Four out of the five breast cancers were of ductal type, and the fifth was lobular histology.

In contrast, the overall risk of cancer was not significantly increased beyond age 50 years (14 cases, SIR=1.40 (0.7–2.4) *P*=0.27) (Table 2b). In this cohort, there were relatively fewer patient-years of follow-up in this age group limiting, the power to detect a significant effect, although the power to detect a two-fold increase in risk was still 75% (at the 5% significance level). There were no brain/CNS tumours beyond this age in the cohort, but the five connective tissue tumours were significantly more than expected in the general population.

Thirty-one of the 36 cancers included in the analysis had been formally confirmed by Cancer Registry report; four of the five cases reported only on death certificates were connective tissue tumours, and the fifth was of unknown site. Excluding these five unconfirmed cases reduced the connective tissue SIR to 77.7 (31.2–160), which was still highly significant (*P*<0.0001). The overall SIR reduced slightly to 2.3 (1.6–3.3, *P*<0.0001) and the SIR for ‘unknown’ cancers was no longer significant (SIR=4.3 (0.9–12.5, *P*=0.07).

Although our analysis was restricted to first malignancies, we noted that three of the patients went on to have a second malignant tumour during the period of follow-up; a rectum adenocarcinoma 1 year after a lower limb malignant neurilemmoma, a peripheral nerve malignant neurilemmoma 5 years after a tonsil carcinoma and an upper limb soft tissue sarcoma 5 months after a thorax soft tissue fibrosarcoma.

The cumulative risk of any malignancy in an NF1 patient by age 50 years was estimated to be 20% (95% CI 14–29%), with a 36% risk by age 70 years (95% CI 27–46%), as compared with a risk of around 18% in the general population of England and Wales. This includes a 14% risk of connective tissue cancers (95% CI 7.8–24%) and a 7.9% risk of brain and central nervous system malignancies (95% CI 3.9–16%, as compared with population risks of around 0.12 and 0.46%, respectively, by age 70 years.

Of the 448 individuals in the cohort, 197 were below the age of 20 years at the beginning of the period of follow-up, enabling us to estimate risks of childhood cancer in NF1 patients. Six cancers were observed in patients younger than 20 years old, giving an SIR of 27.8 for this age group (95% CI=10.1–60.2, *P*<0.0001). This is equivalent to an absolute risk of 7.0% by age 20 years (95% CI=3.2–14.9%), or around 1 in 14, compared to a risk of around 1 in 380 in the general population. Four of the cancers were brain tumours and two were connective tissue cancers (Appendix A).

## DISCUSSION

This prospective study further delineates the risks of malignancy in NF1 patients and will thus provide useful information for clinicians who are advising families in this difficult area. The overall risk of cancer was increased by a factor of 2.7 in these patients, but this was largely owing to marked increases in the risks of brain/CNS and connective tissue tumours. Excess cancer risks were more evident below age 50 years. The data suggesting a possible earlier onset of breast cancer in individuals with NF1 are particularly important, given the possible screening implications.

The main strengths of this study are the large sample size (and hence, high statistical power to detect a modest increase in cancer risk) and the combination of a prospective design with the use of cancer registry information to remove possible recall or reporting biases. Patients with a diagnosis of cancer before their enrolment in the study were excluded, avoiding the possibility that patients who had had a cancer might be more motivated to take part. Recruiting patients via a support group is unlikely to have biased the results, unless NF1 patients who are members of the support group have a different risk of cancer than the wider population of NF1 patients.

Previous reports of malignancy rates in NF1 had varied in their assessment of the risks associated with this condition. In the longitudinal study by Sorensen *et al* (1986), 212 patients with NF1 were followed up over a period of 40 years, and 57 malignant tumours were found in this population. Although many of these tumours were those seen commonly in the general population, there was a greatly increased rate of CNS tumours in the study group, particularly gliomas. The authors acknowledged that their results were limited by the ascertainment method: patients were recruited via hospital clinics and thus may well have had a greater incidence of complications consequent to NF1. It is not clear whether the presence of multiple manifestations of NF1 increases the chance of malignancy for that individual, although the presence of neurofibromas is recognised as a risk factor for malignant peripheral nerve sheath tumours (MPNST) (Evans *et al*, 2002).

Another longitudinal Scandinavian study (Zoller *et al*, 1997) estimated the rates of malignancy in 70 adult patients with NF1. Around 15% of patients in this cohort developed malignant tumours in a 12-year follow-up period, which greatly exceeds our incidence rate for malignant tumours. Their relative risk estimate of 4.0 (95% CI=2.0–7.1) was also greater than our estimate of 2.7 (95% CI=1.9–3.7), with a different range of observed tumour types. In our larger study, we report a preponderance of brain/CNS and connective tissue tumours, whereas although sarcomas were reported in 7% of individuals with NF1, this Swedish study reported no individuals with CNS malignancy. The reason for this apparent discrepancy may be that the Swedish cohort only included adult patients, whose mean age at the start of the study was 44 years (range 20–81 years), in contrast to our UK cohort, whose mean age at entry was 27 years (range=7 months to 72 years). We observed only one CNS malignancy after 40 years of age (a malignant neurofibrosarcoma of the spinal cord in a 49-year-old male).

The only other UK-based study of general malignancy in NF1 patients was the cross-sectional population-based study by Huson *et al* (1988). This study was, perhaps, the least likely to be biased towards ascertainment of cancer. The data generated by this study gave a higher CNS malignancy rate than ours, at 4.4%, with an overall malignancy risk of 7%. Another, larger, population-based study in the USA (Friedman and Birch, 1997) gave an overall risk of malignancy of 4.6%, with CNS malignancy risk at 2%. Neither study estimated relative risks for the observed number of malignancies compared to that expected in the general population.

The other approach used to study the risk of cancer in NF1 is to ascertain the number of cases of NF1 in a population of cancer patients. This has been carried out previously by Matsui *et al* (1993) and Baptiste *et al* (1989). Although both these studies verified the increased risk of cancer in NF1, there are problems with this type of study in terms of ascertainment bias.

The other types of study in this area reported in the literature are those involving mortality statistics. An early Japanese study by Imaizumi (1995) found that the average age of death from ‘neurofibromatosis’ was 43 years. One of the major problems with this study in the context of our work is that it included patients with both NF1 and NF2. Given the high incidence of CNS tumours (mostly vestibular schwannomas) in NF2 patients, it is not possible to extrapolate the data in this study to our work.

Another more recent study that of Rasmussen *et al* (2001) found that overall survival in individuals with NF1 is reduced by some 15 years in comparison with that expected in the general population. The most increased relative risk of tumours causing death in this population involved those of connective tissue and other soft tissue, with CNS tumours also causing significant mortality.

Other studies have reported an increased incidence of juvenile myelomonocytic leukaemia (JMML) in children with NF1 (Stiller *et al*, 1994). This malignancy has been reported to have an association with the presence of juvenile xanthogranulomas in this population. We did not encounter any cases of JMML in our study, but as this is an extremely rare form of leukaemia, representing only 1.6% of haematological malignancy in children (Arico *et al*, 1997), this may be owing to insufficient numbers of cases recruited.

Other tumours have been reported as having increased incidence in NF1. These include rhabdomyosarcoma and phaeochromocytoma (Modlin *et al*, 1979; Hartley *et al*, 1988). We encountered one case of rhabdomyosarcoma and one case of paraganglioma. It is difficult to verify the previously reported associations given these small numbers, as these tumours may simply have occurred by chance.

Although we excluded neurofibromas from our figures on overall malignancy risks, these lesions are known to be significant precursors in the development of MPNST or neurofibrosarcomas. More than a third of patients in our study had a reported neurofibroma, and other cases may have existed that were not formally registered, and therefore not reported to the study. Data have previously been presented concerning the risks of MPNST in NF1 (Evans *et al*, 2002), and a lifetime risk of 8–13% in this population has been quoted. This corresponds to the overall 14% (95% CI=7.8–24%) risk of connective tissue tumours by age 70 years estimated in our cohort (the majority of which were neurofibrosarcomas).

Finally, our study shows that the risk of breast cancer was significantly higher in women younger than 50 years of age than in the general population; SIR=4.0 (1.1–10.3), *P*=0.037. Of five cases of breast cancer diagnosed in the cohort, four were before age 50 years, and the fifth case was diagnosed at age 56 years. Although these are small numbers, if further work in this area confirms an increased risk of relatively early-onset breast cancer in patients with NF1, this could have implications for breast screening in this population.

This prospective study of malignancy risk in 448 patients with NF1 has shown substantially increased relative risks for CNS and connective tissue tumours over the general population, contributing to an estimated overall risk of malignancy of 36% (95% CI=27–46%) by age 70 years. This information will be useful in counselling patients in this difficult area, but further work will be required to delineate whether there are subgroups of patients who can be defined molecularly as being at substantially increased risk of malignancy, and whether screening for certain tumours, in particular young onset breast cancer, is warranted in this group of patients.

## Figures and Tables

**Table 1:** Observed and expected numbers of cancers, with standardised incidence ratios, for cancer sites at which at least one case was observed

**Cancer site**	**ICD code (rev 10)**	**Observed**	**Expected**	**SIR**	**95% CI**	***P*-value**
Buccal cavity and pharynx	C00–14	1	0.243	4.12	0.10–23.0	0.43
Oesophagus	C15	1	0.285	3.52	0.09–19.6	0.50
Colorectal	C18–21	2	1.48	1.35	0.16–4.87	0.87
Gall bladder	C23–24	1	0.0551	18.2	0.46–101	0.11
Lung	C33–34	1	1.86	0.54	0.01–3.00	0.89
Connective tissue	C47,49	11	0.0900	122	61.0–219	<0.0001
Breast (female)	C50	5	2.67	1.87	0.61–4.37	0.26
Ovary	C56	1	0.434	2.31	0.06–12.8	0.70
Bladder	C67	1	0.653	1.53	0.04–8.54	0.96
Brain/CNS	C70–72	7	0.310	22.6	9.06–46.5	<0.0001
Other sites^a^		1	0.209	4.79	0.12–26.7	0.38
Unknown		4	0.700	5.71	1.55–14.6	0.01
All sites^b^		36	13.4	2.69	1.88–3.72	<0.0001
All sites:^b^ male		13	5.89	2.21	1.17–3.78	0.015
All sites:^b^ female		23	7.50	3.07	1.95–4.61	<0.0001
All sites other than brain or connective tissue		18	13.0	1.38	0.82–2.18	0.22

IR=standardised incidence ratio, CI=confidence interval.

a The ‘other’ cancer was of the parathyroid gland at age 35 years.

b All cancer sites excluding non-melanoma skin cancer.

**Table 2:** (a) Observed and expected numbers of cancers diagnosed below age 50 years, with SIR, for cancer sites at which at least one case was observed and (b) observed and expected numbers of cancers diagnosed at age 50 years or above, with SIR, for cancer sites at which at least one case was observed

**Cancer site**	**ICD code (rev 10)**	**Observed**	**Expected**	**SIR**	**95% CI**	***P*-value**
(a)						
Buccal cavity and pharynx	C00–14	1	0.070	14.3	0.36–46.5	0.14
Colorectal	C18–21	1	0.201	4.97	0.13–27.7	0.18
Gall bladder	C23–24	1	0.0064	157	3.97–875	0.013
Connective tissue	C47,49	6	0.0453	132	48.6–289	<0.0001
Breast (female)	C50	4	0.994	4.02	1.09–10.3	0.037
Bladder	C67	1	0.0725	13.8	0.35–76.8	0.14
Brain/CNS	C70–72	7	0.147	47.8	19.2–98.4	<0.0001
Other sites^a^		1	0.0669	15.0	0.38–83.3	0.13
All sites^b^		22	3.38	6.51	4.08–9.82	<0.0001
All sites:^b^ male		7	1.13	6.22	2.49–12.8	0.0003
All sites:^b^ female		15	2.26	6.65	3.72–11.0	<0.0001
All sites other than brain or connective tissue		9	3.19	2.82	1.29–5.36	0.011
						
(b)						
Oesophagus	C150	1	0.252	3.97	0.10–22.1	0.45
Colorectal	C18–21	1	1.28	0.781	0.02–4.35	1.00
Lung	C33–34	1	1.69	0.592	0.01–3.29	0.99
Connective tissue	C47,49	5	0.0447	112	36.2–261	<0.0001
Breast (female)	C50	1	1.67	0.599	0.02–3.33	0.81
Ovary	C56	1	0.304	3.30	0.08–18.4	0.52
Unknown		4	0.599	6.68	1.82–17.1	0.0067
All sites^b^		14	10.0	1.40	0.76–2.35	0.27
All sites:^b^ male		6	4.76	1.26	0.46–2.75	0.69
All sites:^b^ female		8	5.24	1.53	0.66–3.01	0.32
All sites other than brain or connective tissue		9	9.80	0.92	0.42–1.75	0.97

IR=standardised incidence ratio, CI=confidence interval.

a The ‘other’ cancer was of the parathyroid gland at age 35 years.

b All cancer sites excluding non-melanoma skin cancer.

**Table A1:** 

**ICD code (rev 10)**	**Age at diagnosis**	**Sex**	**Description of tumour**
C09.9	47	F	Tonsil squamous cell carcinoma
C15.9	72	M	Oesophagus adenocarcinoma
C18.4	53	F	Transverse colon adenocarcinoma
C20.0	40	F	Adenocarcinoma of rectum
C24.1	37	M	Carcinoma of Ampulla of Vater
C34.2	75	M	Squamous cell carcinoma of lung
C47.2	25	F	Lower limb neurofibrosarcoma
C47.1	69	M	Lower limb malignant neurilemmoma/schwannoma
C47.3	47	F	Thoracic peripheral nerve neurofibrosarcoma
C47.3	53	F	Malignant tumour of peripheral nerves (thorax)^a^
C47.3	47	M	Spindle cell sarcoma of thoracic peripheral nerve
C47.9	55	M	Small bowel neurofibrosarcoma
C47.9	50	M	Malignant peripheral nerve tumour^a^
C47.9	14	F	Neurofibrosarcoma^a^
C49.1	70	M	Upper limb myxoid chondrosarcoma
C49.3	19	F	Thorax soft tissue fibrosarcoma
C49.9	22	M	Fibrosarcoma^a^
C50.9	48	F	Breast infiltrating duct carcinoma
C50.9	56	F	Breast ductal carcinoma
C50.9	39	F	Breast ductal carcinoma
C50.9	46	F	Lobular breast carcinoma
C50.9	43	F	Ductal breast carcinoma
C56.0	55	F	Ovarian carcinoma
C67.9	49	F	Bladder transitional cell carcinoma
C71.9	39	F	Brain (unspecified) astrocytoma
C71.9	17	M	Brain malignant neoplasm
C71.9	4	F	Brain (unspecified) astrocytoma
C72.0	49	M	Malignant neurofibrosarcoma of spinal cord
C72.5	30	M	CNS neurofibrosarcoma
C72.5	8	M	Cranial nerve malignant glioma
C72.9	7	F	CNS glioma
C74.1	35	F	Adrenal gland malignant neoplasm
C80.0	62	F	Metastatic adenocarcinoma (unspecified site)
C80.0	64	F	Metastatic adenocarcinoma (unspecified primary)^a^
C80.0	73	F	Metastatic adenocarcinoma (unspecified primary)
C80.0	51	F	Carcinomatosis (unspecified primary)

a Cancer report obtained from death certificate, not reported by Cancer Registry.

## Appendix A

List of the 36 cancers used in this analysis (Table A1)
